# Type VI Secretion Systems in *Salmonella* Encode New Effectors with Putative Antibacterial and Anti-Eukaryotic Activities

**DOI:** 10.3390/microorganisms14061232

**Published:** 2026-05-30

**Authors:** Ayleen Parra-Calisto, Carlos A. Santiviago, Carlos J. Blondel, Carla Vargas-del Río, Valentina Briceño, Andrea Avilés, Fernanda Salazar-Salas, Patricio Espinoza-Jara, María J. Faúndez, Dácil Rivera, Andrea Moreno-Switt, Fernando A. Amaya, Leonardo Pavez, David Pezoa

**Affiliations:** 1 Department of Chemical Engineering, Biotechnology and Materials, Centre for Biotechnology and Bioengineering (CeBiB), Universidad de Chile, Santiago 8370456, Chile; ayleenparra@ug.uchile.cl; 2 Núcleo de Microbiología Traslacional para la Vigilancia e Innovación en Sistemas Sanitarios, Ambientales y Productivos MICRA, Facultad de Medicina Veterinaria y Agronomía, Universidad de Las Américas, Campus Providencia, Manuel Montt 948, Santiago 7500975, Chilevalentina.briceno.e@gmail.com (V.B.); patricio.espinoza.jara@edu.udla.cl (P.E.-J.); cotefaundezf@gmail.com (M.J.F.); drivera@udla.cl (D.R.); 3 Laboratorio de Microbiología, Departamento de Bioquímica y Biología Molecular, Facultad de Ciencias Químicas y Farmacéuticas, Universidad de Chile, Santiago 8370456, Chile; csantiviago@ciq.uchile.cl (C.A.S.); andrea.aviles@ug.uchile.cl (A.A.); fernando.amaya@ug.uchile.cl (F.A.A.); 4 Institute of Biomedical Sciences, Faculty of Medicine, Universidad Andres Bello, Santiago 8370071, Chile; carlos.blondel@unab.cl; 5 Escuela de Medicina, Facultad de Salud, Universidad del Alba, Santiago 8320000, Chile; fernandasal.salas@gmail.com; 6 Escuela de Medicina Veterinaria, Facultad de Agronomía e Ingeniería Forestal, Facultad de Ciencias Biológicas y Facultad de Medicina, Pontificia Universidad Católica de Chile, Santiago 8320000, Chile; andrea.moreno@uc.cl; 7 Núcleo de Investigaciones en Ciencias Biológicas, Facultad de Medicina Veterinaria y Agronomía, Universidad de Las Américas, Santiago 7500972, Chile; lpavez@udla.cl; 8 Departamento de Ciencias Químicas y Biológicas, Universidad Bernardo O’Higgins, Santiago 8370993, Chile

**Keywords:** *Salmonella*, T6SS, effector, immunity protein

## Abstract

The type VI secretion system (T6SS) is a contact-dependent, contractile multiprotein complex widely distributed among Gram-negative bacteria. It mediates the translocation of effector proteins into bacterial competitors and eukaryotic host cells, promoting environmental fitness and contributing to virulence. In *Salmonella*, five pathogenicity islands encoding T6SSs (SPI-6, SPI-19, SPI-20, SPI-21, and SPI-22) have been described, along with an expanding repertoire of associated effector proteins. However, their global diversity and distribution remain incompletely resolved due to limited genomic sampling. To address this, we analyzed a curated dataset of 490 *Salmonella* genomes representing 45 serotypes. T6SS regions were identified using SecreT6, revealing that SPI-6 is widely distributed, whereas SPI-19, SPI-20, and SPI-21 are restricted to a subset of serotypes. SPI-20 and SPI-21 were exclusively found in *S. enterica* subsp. *arizonae* and *diarizonae*, while SPI-22 was absent from all analyzed genomes. All open reading frames within T6SS clusters were then analyzed for effector prediction and functional annotation. This approach recovered 32 out of 45 previously described T6SS effectors and identified several novel candidates. These included a cytidine deaminase with predicted DNase activity in SPI-6: two candidate nuclease effectors in SPI-19 with DNase and RNase activities, and four putative effectors in SPI-21, including enzymes with predicted peptidoglycan hydrolase activity, a potential inhibitor of eukaryotic ATPases, and a membrane pore-forming toxin. Additionally, a putative phospholipase effector was identified within a VgrG-associated genomic island in a subset of *S. enterica* subsp. *diarizonae* isolates. Collectively, these findings expand the known repertoire of *Salmonella* T6SS effector proteins and highlight their functional diversity.

## 1. Introduction

The type VI secretion system (T6SS) is a contractile protein apparatus that translocates effector proteins into target prokaryotic and/or eukaryotic cells [1]. These effectors display a high level of diversity conferring remarkable functional versatility, targeting essential molecules of recipient cells [2,3]. Effector proteins with antibacterial activity, which are encoded in bicistronic elements with their immunity proteins to prevent self-intoxication [2], target the peptidoglycan [2,4,5,6] or the FtsZ division ring [7]. Another T6SS effector targets the actin filaments or microtubule network of eukaryotic cells [3], while another group called trans-kingdom effectors, affects energetic cofactors, nucleic acids, phospholipids, and membranes of both bacteria and eukaryotic cells [8,9,10].

In *Salmonella*, five T6SS gene clusters have been identified within SPI-6, SPI-19, SPI-20, SPI-21, and SPI-22 [11,12] that are diversely distributed across *Salmonella* serotypes, subspecies, and species, indicating a heterogeneous evolutionary and ecological pattern [11].

**Figure 1:**
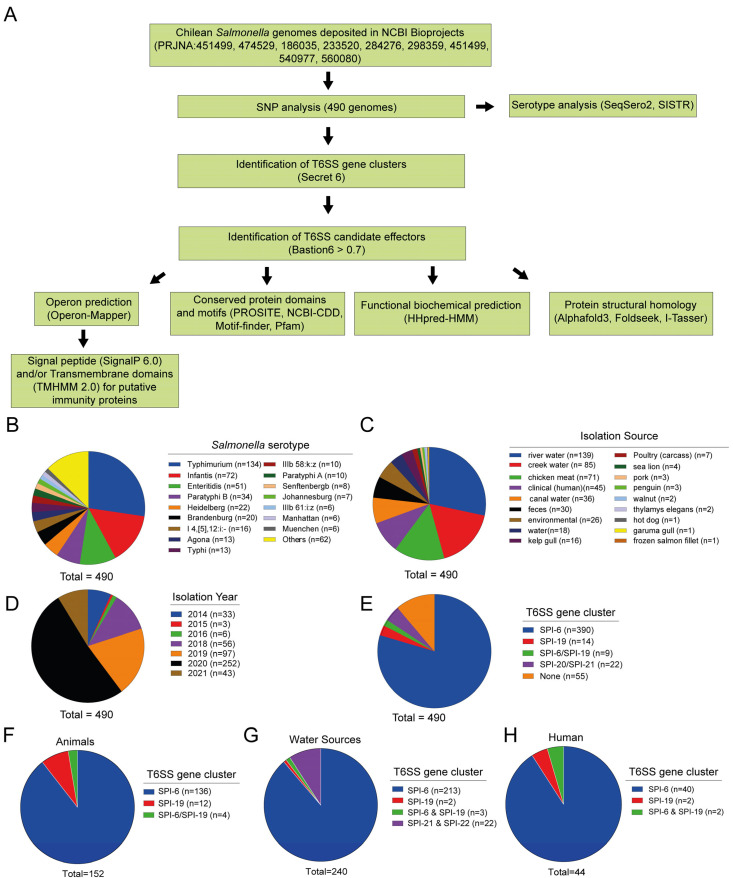
Study design and summary of the Chilean local *Salmonella* genome database. (**A**) Flowchart of the identification of T6SS gene clusters and their associated candidate effectors in 490 high-quality Chilean *Salmonella* genomes obtained from the NCBI platform. (**B**–**E**) Isolates were grouped by (**B**) serotypes, (**C**) isolation source, (**D**) time period, (**E**) T6SS gene cluster identified and T6SS gene cluster per isolation source (**F**–**H**).

Despite their widespread distribution, the functional roles of T6SSs in *Salmonella* remain incompletely understood. Most current knowledge derives from studies of the SPI-6 and SPI-19 T6SSs, which have implicated these systems in interbacterial competition, virulence, and host interactions [13,14,15,16,17,18]. In parallel, the repertoire of T6SS effectors has expanded considerably in recent years through genomic and bioinformatic analyses [19,20]. Nevertheless, the global set of T6SS effectors across *Salmonella* serotypes remains far from fully characterized. To date, several validated and putative effector proteins have been identified in *Salmonella* that target diverse bacterial components including peptidoglycan, nucleic acids, membranes, and ribosomes [13,14,15,16,18,21]; however, these findings are restricted to a limited subset of serotypes. This gap in knowledge is significant, as effector proteins ultimately determine T6SS function and specificity. Given the extensive diversity observed even within a small subset of serotypes, comprehensive identification and characterization of T6SS effectors across all T6SS-positive *Salmonella* serotypes is essential to better understand their roles in pathogenesis and environmental adaptation. Recent large-scale bioinformatic efforts, including analyses of genomes from the 10K *Salmonella* database, have identified numerous candidate effectors harboring putative antibacterial domains, thereby substantially expanding the known diversity of T6SS-associated toxins [20]. These studies provide a valuable framework for future large-scale, comparative analyses. Consistent with this, our recent investigation of Chilean *Salmonella* isolates from environmental and animal sources demonstrated that T6SS gene clusters are widespread and encode a diverse array of candidate antibacterial effectors [22], although current knowledge remains limited in both scope and resolution. Accordingly, investigating the global diversity of T6SS effectors requires comprehensive analyses that incorporate large, geographically diverse genomic datasets. In this study, we sought to expand systematic approaches for identifying T6SS gene clusters and their associated effector repertoires by conducting integrated bioinformatic, comparative genomic, and structural prediction analyses using a curated collection of 490 *Salmonella enterica* genomes encompassing 45 serotypes derived from diverse sources and temporal contexts in Chile (Figure 1).

## 2. Materials and Methods

### 2.1. Identification of T6SS Gene Clusters

*Salmonella* genomes isolated in Chile were recovered from the NCBI, including those available under the BioProjects PRJNA451499, PRJNA474529, PRJNA186035 (Mexico), PRJNA233520, PRJNA284726, PRJNA298359, PRJNA540977, and PRJNA560080.

SeqSero [23] and SISTR [24] analyses were performed for in silico serotyping of *Salmonella* isolates. To evaluate clonality SNP analysis was carried out. Isolates diverging by ≤20 SNPs were classified as clonal, in accordance with a previous publication [25]. For downstream analyses, only one representative genome from each clonal group was retained. The final dataset comprised 490 *Salmonella enterica* genomes representing 32 different serotypes (Supplementary Table S1).

T6SS-encoding regions were recognized by means of the SecReT6 server [26], focusing on clusters encoding the 13 core structural components. BLASTp (v2.10.1+) thresholds of >30% sequence identity and an E-value < 0.0001 were applied to define positive matches, as previously validated for T6SS identification in *Salmonella* (Figure 1A) [27,28].

### 2.2. Identification of Candidate T6SS Effectors

Open reading frames (ORFs) located within SPI-6 were systematically screened for putative T6SS effector proteins using Bastion6 [29]. Predicted effectors were further analyzed using Operon-Mapper [30] to evaluate their organization within bicistronic units potentially encoding cognate immunity proteins. Immunity proteins were defined as small proteins harboring signal peptides and/or transmembrane domains, predicted using SignalP 6.0 and TMHMM 2.0.

Functional annotation of candidate proteins was performed using domain and motif searches against PROSITE, NCBI Conserved Domain Database, Motif Finder, and Pfam via the GenomeNet, applying an E-value cutoff of 0.01 [31,32,33,34]. Functional predictions were further refined using HMM–HMM comparisons with HHpred [35] (Figure 1A).

Given the lack of standardized nomenclature for many ORFs, candidate effector and immunity proteins are referred to according to previously reported names, or when novel, based on their predicted functional domains.

Finally, a candidate T6SS effector was classified as “novel” when it satisfied at least one of the following criteria: (i) the presence of at least one domain previously associated with antibacterial/anti-eukaryotic activity; (ii) a predicted three-dimensional structure exhibiting high similarity to that of a characterized antibacterial/anti-eukaryotic protein; and/or (iii) the inclusion of a protein domain not previously reported as part of a T6SS effector in publicly available databases.

### 2.3. Computational Structural Characterization and Homology Modeling of Candidate T6SS Effector Proteins

Protein structures were inferred by AlphaFold3 analyses under default parameters [36]. The resulting models were evaluated and selected based on optimal scores. Quality metrics such as ipTM and pTM are reported within the figures and metrics such as pLDDT are reported in Supplementary Figure S1. Structural homologs were identified using Foldseek [37]. Visualization, structural alignment, and superimposition analyses were carried out on PyMOL (v3.1) and the MatchMaker tool implemented in UCSF ChimeraX v 1.11.1 [38]. The models are available in ModelArchive (modelarchive.org) with the accession codes ma-kpdxp (QS27_14110-CT with QS27_14115), ma-05nwf (QS34_17685 with QS34_17680), ma-tg4ob (GI454_16755 with GI454_16750), and ma-ayfcj (JHT71_003331 with JHT71_003332).

### 2.4. Sequence and Phylogenetic Analyses

Nucleotide sequences encoding the identified T6SS effectors were employed as queries in BLASTn searches against all *Salmonella* genomes from the NCBI (accessed October 2025). Orthologous sequences were defined based on thresholds of ≥90% sequence identity and ≥90% query coverage. Sequence conservation was evaluated by T-Coffee Expresso [39] and MAFFT v 7.0 [40] sequence alignment analyses and subsequently visualized with ESPript v 3.2 [41]. Comparative genomic of T6SS gene clusters were carried out by means of Mauve [42] and EasyFig (v2.2.5) [43]. Finally, nucleotide sequence inspection and annotation were performed using Artemis (version 18) [44].

## 3. Results

### 3.1. Distribution of Salmonella T6SS Gene Clusters

We analyzed 490 *Salmonella* genomes from isolates obtained from surface water, human, and animals (Figure 1; Supplementary Table S1). The dataset was predominantly composed of environmental isolates, with 278 genomes originating from surface waters representing 27 serotypes (Figure 1C). Animal-derived isolates included 115 genomes corresponding to 14 serotypes, obtained from domestic species (chickens, horses, pigs, and salmon) (Figure 1C). Additionally, 37 isolates representing 16 serotypes were recovered from wild animals including kelp gulls, sea lions, garuma gulls, and penguins. Human clinical isolates accounted for 47 genomes spanning nine serotypes (Figure 1C) while the source of one isolate was not reported. The most prevalent serotypes identified were *S.* Typhimurium (*n* = 134), *S.* Infantis (*n* = 72), and *S.* Enteritidis (*n* = 51) (Figure 1B; Table S1).

We identified 463 putative T6SS gene clusters across 432 genomes (Figure 1B–E; Table S1), while no T6SS gene cluster associated with SPI-22 was detected in any of the analyzed genomes. Among the identified loci, SPI-6 was the most prevalent, occurring in 399 out of 490 genomes (81.43%). In contrast, SPI-19, SPI-20, and SPI-21 were detected at markedly lower frequencies, present in 23 (4.7%), 22 (4.5%), and 22 (4.5%) genomes, respectively (Figure 1E; Table S1). The majority of isolates harbored a single T6SS cluster, most commonly SPI-6 or SPI-19. Notably, isolates assigned to *S.* Dublin carried both SPI-6 and SPI-19 clusters, whereas SPI-20 and SPI-21 were exclusively identified in *S.* arizonae, with most strains containing both loci (Table S1).

### 3.2. Identification of a Novel SPI-6-Encoded Effector/Immunity Module with Predicted Nuclease Activity

To identify high-confidence T6SS effectors, all ORFs within detected clusters were evaluated using a multi-criteria approach, including Bastion6 prediction, operon structure analysis (Operon-mapper) [30], signal peptide and transmembrane domain prediction (SignalP 6.0 and TMHMM 2.0), identification of conserved domains (InterProScan, PROSITE, Pfam, MOTIF, and NCBI-CDD), and structural homology inference using HHpred. Additional analyses were conducted to identify previously unannotated ORFs.

**Figure 2:**
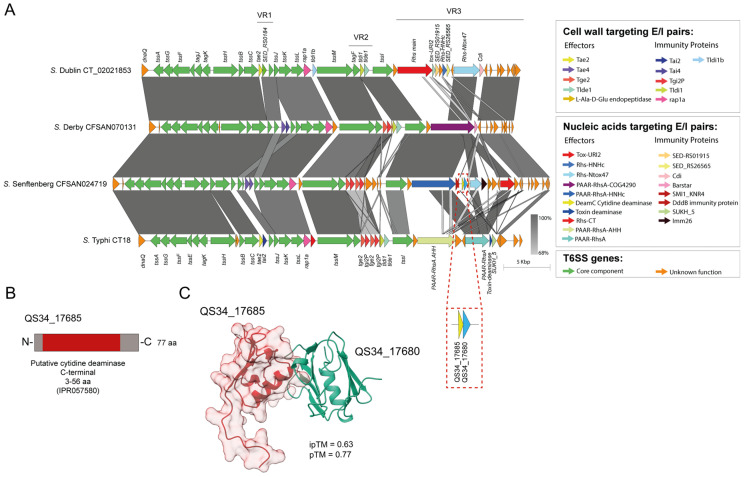
A new putative effector/immunity (E/I) module with predicted nuclease activity is encoded in SPI-6. (**A**) Genomic analysis of the *S.* Senftenberg CFSAN024719 SPI-6 in comparison with *S.* Dublin CT_02021853, *S.* Derby CFAN070131, and *S.* Typhi CT18. ORFs corresponding to E/I modules are color-coded associated with their experimentally validated or predicted functions, while grayscale shading indicates nucleotide sequence identity. (**B**) Schematic representation of the candidate effector QS34_17685 and its putative cognate immunity protein QS34_17680 from *S.* Senftenberg CFSAN024719. (**C**) Complex structure of the QS34_17685/QS34_17680 T6SS E/I pair.

Depending on the serotype, the SPI-6 T6SS gene cluster spans approximately 35–50 kb and encodes ~30–45 open reading frames (ORFs), including the full complement of 13 core T6SS components. Although the overall genetic architecture of the SPI-6 T6SS cluster is highly conserved among serotypes, structural variability is confined to three discrete regions of the island designated as variable regions (VR1, VR2, and VR3) [11,28]. In the variable region 3 of SPI-6 (located between *tssI* gene and fimbrial operons (e.g., *saf*)) from *S.* Senftenberg isolates, we identified a new E/I module (Figure 2; Table 1). The candidate effector (QS34_17685) is a 77-amino acid protein predicted to exhibit DNase activity and contains a putative cytidine deaminase domain (IPR057580) (Figure 2A–C and Table 1). Structural comparison using Foldseek revealed low similarity to the DddB dsDNA deaminase from *Taylorella equigenitalis* (E value 4.27 × 10^−1^). The effector gene is likely part of a same bicistron with a gene that lacks both signal peptides and transmembrane domains, consistent with an immunity protein with cytoplasmic localization (Table 1). Alphafold-multimer analysis supported the formation of an E/I complex, with an ipTM score of 0.633 (Figure 2C).

### 3.3. SPI-19 T6SS Gene Cluster Encodes Two Novel Effector/Immunity Modules with Predicted DNase Activity

**Figure 3:**
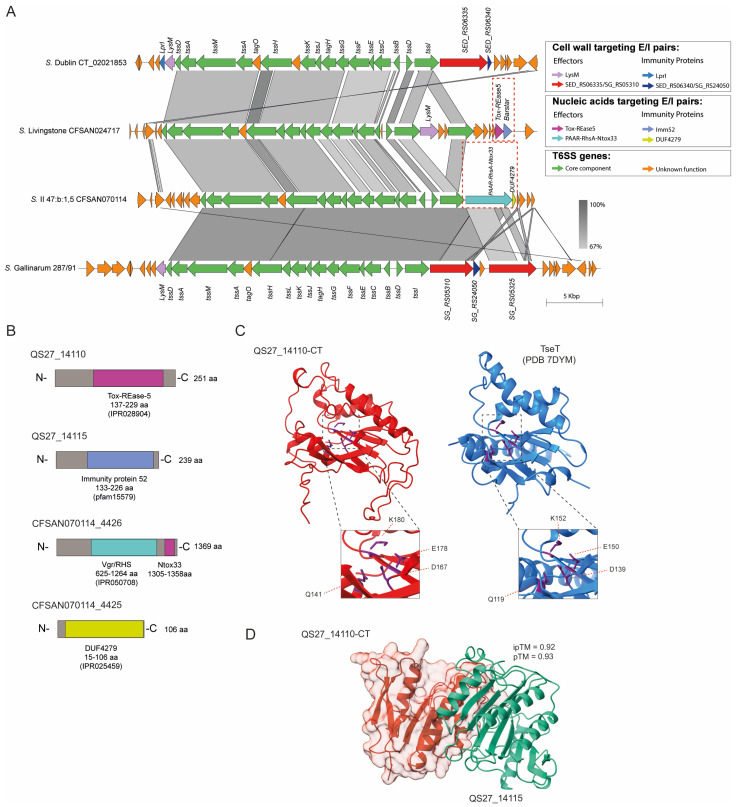
Two new putative effector/immunity (E/I) modules with predicted nuclease activity are encoded within SPI-19. (**A**) Genomic analysis of SPI-19 from *S.* Livingstone CFSAN024717 and *S. enterica* subsp. *diarizonae* II 47:b:1,5 CFSAN070114 in comparison with *S.* Dublin CT_02021853 and *S.* Gallinarum 287/91. ORFs encoding E/I modules are color-coded associated with their confirmed or predicted functions, while grayscale shading indicates nucleotide sequence identity. (**B**) Schematic representation of the candidate effectors QS27_14110 and CFSAN070114_4426 and their cognate putative immunity proteins, QS27_14115 and CFSAN070114_4425, identified in *S.* Livingstone CFSAN024717 and *S. enterica* subsp. *diarizonae* II 47:b:1,5 CFSAN070114, respectively. (**C**) Structural comparison between the AlphaFold3-predicted model of QS27_14110 and the experimentally resolved structure of the TseT DNase effector from *Pseudomonas aeruginosa* (PDB: 7dymA). The conserved catalytic QDE triad is highlighted in both structures. (**D**) Complex structure of the QS27_14110CT/QS27_14115 T6SS E/I pair.

The T6SS encoded in SPI-19 was identified in 23 isolates. Two novel candidate effectors with predicted nuclease activity were detected (Table 2; Figure 3).

The first effector (QS27_14110), identified in *S.* Livingstone, is a protein of 251-amino acid containing a Tox-REase-5 domain (IPR028904) (Table 2; Figure 3A–C). Structural analyses using Foldseek and I-TASSER revealed homology to the TseT DNase effector of *Pseudomonas aeruginosa* (PDB 7DYM) [45], including conservation of the catalytic triad, strongly supporting DNase activity (Figure 3C). This gene is part of a bicistron with QS27_14115 that encodes a protein containing an Immunity protein 52 domain (pfam15579) (E-value 3.21 × 10^−18^) (Table 2), commonly associated with DNase toxin systems [46] (Figure 3B). Alphafold-multimer analysis indicated a high-confidence interaction (ipTM = 0.92), consistent with an E/I pair (Figure 3D). The prevalence and distribution of the Tox-Rease-5 effector across the different serovars are summarized in Supplementary Table S2.

The second candidate effector (CFSAN070114_4426) is a Rhs protein of 1369 amino acids. This protein carries an additional putative Ntox33 domain in its C-terminal end (Figure 3B). This gene forms a bicistronic unit with CFSAN070114_4425, encoding a DUF4279-containing protein likely functioning as its cognate immunity protein (Figure 3A and Table 2). Notably, DUF4279 domains (IPR025459) have recently been associated with immunity proteins in polymorphic toxin systems [47].

### 3.4. SPI-21 T6SS Gene Cluster Encodes Two Antibacterial and Two Anti-Eukaryotic Candidate Effectors

**Figure 4:**
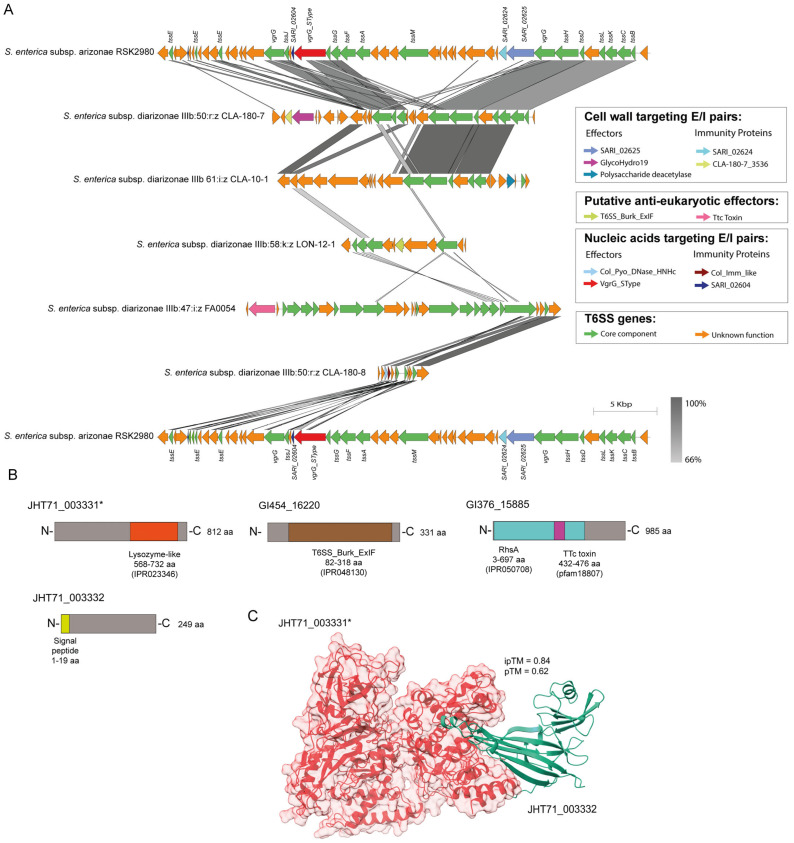
Two putative antibacterial effectors and two putative antieukaryotic effectors are encoded by SPI-21. (**A**) Genomic analysis of SPI-21 from *S. enterica* subsp. *diarizonae* strains IIIb:50:r:z CLA-180-7, IIIb:61:i:z CLA-10-1, IIIb:58:k:z LON-12-1, IIIb:47:i:z FA0054, and IIIb:50:r:z CLA180-8 in comparison with *S. enterica* subsp. *arizonae* strain RSK2980. Open reading frames (ORFs) encoding effector/immunity (E/I) modules are color-coded associated with their experimentally validated or predicted functions, while grayscale shading indicates nucleotide sequence identity. (**B**) Schematic representation of two predicted antibacterial effectors, JHT71_003331 and CLA-10-1_4787—putative peptidoglycan hydrolases—from *S. enterica* subsp. *diarizonae* strains IIIb:50:r:z CLA-180-7 and IIIb:61:i:z CLA-10-1, respectively, along with their cognate putative immunity proteins. In addition, two predicted antieukaryotic effectors, GI454_16220 and GI376_15885, identified in strains IIIb:58:k:z LON-12-1 and IIIb:47:i:z FA0054, respectively, are shown. (**C**) Predicted protein–protein complex structure of the JHT71_003331/JHT71_003332 T6SS effector/immunity pair generated using AlphaFold3, with the corresponding interface predicted TM-score (ipTM) indicated. * corresponds to truncated version of the protein.

Analysis of the region encoding the T6SS of SPI-21 identified four candidate effectors (Table 3; Figure 4A–C). One effector (JHT71_003331) contains a lysozyme-like domain (IPR023346), suggesting peptidoglycan hydrolase activity (Figure 4A,B), while another (CLA-10-1_4787) harbors a polysaccharide deacetylase PgdA-like domain (IPR037950). JHT71_003331 is encoded in a bicistronic unit with a predicted immunity protein (JHT71_003332). This latter ORF encodes a 249 amino acid protein with a Sec/SPI signal peptide and no predicted functional domains that may correspond to its cognate immunity protein (Table 3 and Figure 4B). Of note, structural modeling supports that JHT71_003331/JHT71_003332 forms a protein–protein complex (ipTM = 0.84) (Figure 4C).

Additionally, a candidate effector (GI454_16220) containing a T6SS_ExIF-like domain—an uncharacterized effector family—was identified (Table 4 and Figure 4B). The absence of a cognate immunity protein suggests a potential anti-eukaryotic role. Structural analyses revealed that GI454_16220 shares a low structural similarity to the SidK effector of *Legionella pneumophila*, which inhibits the eukaryotic V-ATPase and reduces phagosomal acidification, thereby promoting intracellular survival [48].

Another effector (GI376_15885) corresponds to a large RhsA protein with a predicted Ttc toxin domain (Figure 4B) similar to toxin complexes of bacterial pathogens that create a pore in the host membrane for translocating toxic enzymes into the host cell [49] (Table 4 and Figure 4B). Although no canonical domains were identified in its C-terminal region, structural similarity to adenylate cyclase toxins (PDB 1K90) was observed at low confidence (Evalue 4.23 × 10^−6^). This effector also lacks an associated immunity protein, further supporting a potential role in host-targeting activity.

### 3.5. Identification of a Novel VgrG-Associated Island Encoding a Phospholipase Effector

**Figure 5:**
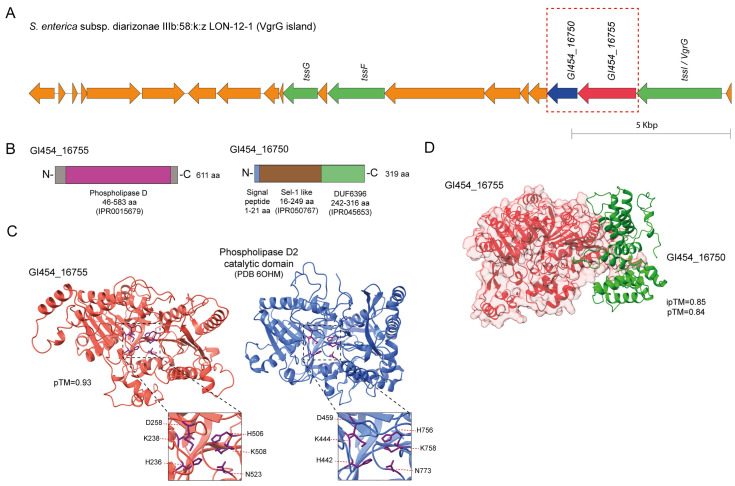
A new VgrG island identified in some strains of *S*. *enterica* subsp. *diarizonae* encodes a new putative effector with predicted phospholipase activity. (**A**) Visualization of the VgrG island identified, highlighting the genetic context of a putative phospholipase and its cognate immunity protein. ORFs encoding phospholipase D/TRP_DUF6396 E/I module are highlighted in red and blue colors, respectively. (**B**) Schematic representation of candidate effector GI454_16755 and its putative immunity protein GI454_16750 from *S*. *enterica* subsp. *diarizonae* IIIb:58:k:z LON-12-1. (**C**) The predicted structure of GI454_16755, generated using AlphaFold3, is shown alongside the crystal structure of human phospholipase D2 (PDB ID: 6OHM_A). Catalytic residues in both proteins are highlighted in purple to emphasize their structural correspondence. (**D**) The AlphaFold3-predicted protein–protein complex between GI454_16755 and GI454_16750, representing a T6SS effector/immunity (E/I) pair, is presented together with the corresponding interface predicted TM-score (ipTM).

A previously uncharacterized VgrG island (~22.2 kb) was identified in *S. enterica* subsp. *diarizonae* isolates (Figure 5). This region encodes approximately 21 ORFs including core T6SS components (tssI/VgrG, tssF, and tssG). Downstream of tssI, a gene (*GI454_16755*) encodes a protein with a predicted phospholipase D domain (IPR015679) (Table 3 and Figure 5). Structural analysis revealed high similarity to human phospholipase D2 (PDB 6ohmA) (Figure 5C).

This gene is co-transcribed with GI454_16750, encoding a protein containing the Sel-1 and DUF6396 domains (Table 3 and Figure 5A,B). Alphafold-multimer analysis supported their interaction (ipTM = 0.82), consistent with a T6SS E/I module (Figure 5D).

### 3.6. Distribution of Newly Identified T6SS Effectors Across Salmonella

To assess the distribution of the eight newly identified candidate effectors, tBLASTx searches were performed against publicly available *Salmonella* genomes (NCBI, December 2025). The analysis revealed a restricted distribution across serotypes (Table S2). The SPI-6-encoded cytidine deaminase effector was detected in five serotypes, whereas SPI-19-associated effectors were present in 10–14 serotypes.

Among the SPI-21 effectors, three (lysozyme-like, T6SS_ExIF-like, and Ttc toxin) were found to be present only in *Salmonella enterica* subsp. *arizonae* and *Salmonella enterica* subsp. *diarizonae*. In contrast, the PgdA-like effector exhibited a broader distribution, being identified in 110 serotypes (Table S2).

## 4. Discussion

The T6SS is a critical determinant of pathogenesis and adaptation in numerous Gram-negative bacteria, translocating a diverse repertoire of effector proteins. As such, it represents a central mechanism underlying interbacterial competition and host–pathogen interactions. In *Salmonella*, the overall prevalence, distribution, and effector repertoire of T6SS clusters remain incompletely characterized. This limitation is partly attributable to the restricted size and diversity of genome datasets analyzed to date [20,22]. Accordingly, to study the global diversity of T6SS effectors, comprehensive analyses incorporating larger and geographically diverse genomic datasets are necessary.

In the present study, we conducted an expanded analysis of T6SS gene clusters and their associated effector repertoires by leveraging a comprehensive collection of *Salmonella* genomes derived from diverse sources and temporal frameworks in Chile. Consistent with previous reports, the T6SS encoded within SPI-6 emerged as the most prevalent cluster [20,22]. In contrast, SPI-19 was confined to a few subsets of serotypes. This restricted distribution suggests a potential role for T6SS_SPI-19 in adaptation to specific ecological niches or host environments. Furthermore, SPI-20 and SPI-21 were exclusively detected in *S. arizonae* and *S. diarizonae*, in agreement with previous observations [11,50]. The SPI-22 cluster was not identified in this dataset, consistent with its restriction to *S. bongori*, which was not represented among the analyzed genomes.

With respect to the effector repertoire, 32 out of the 45 previously described *Salmonella* T6SS effectors were identified among the Chilean isolates analyzed. Furthermore, we report a new putative effector predicted to possess nuclease activity, located within VR3, of SPI-6 underscoring the extensive diversity of T6SS effectors targeting bacterial DNA [19,20,22,27,28]. Notably, nucleic acids appear to represent primary targets of SPI-6-associated effectors, which are predominantly encoded within VR3. Within this region, we identified a new cytidine deaminase (DeamC family) homologous to DddB (BadTF3) from *Taylorella equigenitalis*, thereby expanding the repertoire of predicted deaminase toxins alongside previously reported domains such as Tox-deaminase, STox_20, and Tox-CNF [20,28], suggesting a role in interbacterial competition. Cytidine deaminases catalyze the conversion of cytidine into uridine [51], inducing DNA hypermutation, alteration of gene expression, or inducing antiviral defense mechanisms [52]. Collectively, these observations reinforce the notion that SPI-6 harbors a diverse arsenal of effectors with putative antibacterial activity, including previously uncharacterized candidates, underscoring its importance in *Salmonella* ecological success.

In contrast to our previous analysis of Chilean *Salmonella* isolates [22], here we detected two additional candidate effectors with predicted nuclease activity (Tox-Rease-5 and PAAR-RhsA-Ntox33), one of which is associated with an Rhs protein. These findings further support the role of Rhs elements in diversifying T6SS effector functions. Interestingly, these proteins are arranged in YD-repeat regions that fold into β-cage structures capable of encapsulating and protecting C-terminal toxin domains [53,54], providing a structural basis for their frequent association with T6SS effectors.

Consistent with previous reports, SPI-21 encodes candidate effectors targeting diverse bacterial components, including peptidoglycan hydrolases, pore-forming toxins, and nucleases, each typically associated with cognate immunity proteins [11,20,22,55]. However, only a subset of these effectors has been experimentally validated [55]. Notably, we identified two novel candidate effectors that exhibit structural similarity to previously characterized anti-eukaryotic proteins and lack associated predicted immunity proteins, suggesting that they may function as anti-eukaryotic effectors. One of these contains a T6SS_Burk_ExIF domain with low structural similarity to the SidK effector of *Legionella pneumophila*, which inhibits the eukaryotic V-ATPase and reduces phagosomal acidification, thereby promoting intracellular survival [48]. This raises the possibility that SPI-21-encoded T6SS may contribute to intracellular persistence within macrophages. The second candidate effector harbors a Ttc toxin domain, typically associated with tripartite Tc toxin complexes that form pores in host cell membranes, facilitating the translocation of toxic enzymes. Together, our results suggest that the T6SS encoded in SPI-21 may play dual roles in antibacterial activity and host interaction.

Additionally, we identified a VgrG-associated genomic island in a subset of *S. enterica* subsp. *diarizonae* serotype IIIb 58:k:z isolates, encoding a novel candidate effector with predicted phospholipase D activity. This finding expands the known repertoire of membrane-targeting mechanisms employed by *Salmonella* T6SS effectors and suggests a role for this VgrG island in interbacterial competition. In addition, these T6SS putative effectors were distributed among a limited number of serotypes, consistent with previous observations [22,28]. Of note, some of the domains present in our new effectors identified (such as Tox-Rease-5, Ntox33 and Ttc toxin) have been previously reported [20]; however, their genomic localization, genetic context, and predicted structures were not defined, and thus it remains unclear whether they correspond to the effectors identified in this study. Overall, this study expands the known repertoire of *Salmonella* T6SS effector proteins and demonstrates that the SPI-6, SPI-19, SPI-20, and SPI-21 gene clusters encode a diverse array of putative antibacterial effectors. Although our findings substantially increase the number of predicted effectors targeting competing bacteria, we cannot exclude the possibility that several newly identified candidates—particularly those predicted to act on nucleic acids, ATPases, and cellular membranes—may also exert activity against eukaryotic cells. This uncertainty highlights an important gap in our current understanding of the contribution of T6SS to host–pathogen interactions.

## 5. Conclusions

In this study, we extend the systematic identification and distribution analysis of T6SS gene clusters in a collection of 490 *Salmonella enterica* genomes, derived from diverse sources and temporal contexts in Chile, demonstrating that SPI-6 is broadly distributed, whereas SPI-19, SPI-20, and SPI-21 are confined to a subset of serotypes. Furthermore, our approach recovered 32 out of the 45 previously described T6SS effectors and uncovered several novel candidates. These include a cytidine deaminase with predicted DNase activity within SPI-6; two putative nuclease effectors in SPI-19 with predicted DNase and RNase activities; and four candidate effectors in SPI-21, comprising enzymes with predicted peptidoglycan hydrolase activity, a potential inhibitor of eukaryotic ATPases, and a membrane pore-forming toxin. In addition, we identified a putative phospholipase effector encoded within a VgrG-associated genomic island in a subset of *S. enterica* subsp. *diarizonae* isolates. Collectively, this study broadens the currently *Salmonella* T6SS effectors with putative antibacterial activity. Furthermore, these findings offer novel insights into the potential mechanisms by which T6SS proteins may affect eukaryotic cells, thereby helping to address a longstanding gap in the understanding of *Salmonella* host–pathogen interactions. Further experimental studies will be required to validate these predicted functions and to elucidate the biological roles of these effectors in both interbacterial competition and host infection.

## Figures and Tables

**Table 1:** Identification of a new putative T6SS E/I module encoded within SPI-6.

Gene-Encoding T6SS Effector					Gene-Encoding Immunity Protein	
Gene(s)	Number of aa	Serotype-Isolate	VR	Molecular Target/Domain	Gene(s)	Transmembrane or Signal Peptide/Domain ^a^
Effector That Target Nucleic Acids						
QS34_17685	77	*S*. Senftenberg CFSAN024719	3	DNA/DeamC_Cytidine_deaminase	QS34_17680	No/DdB IP

^a^ This column indicates whether a predicted immunity protein harbors transmembrane domains (TM regions), signal peptides, or conserved protein domains.

**Table 2:** Identification of new putative T6SS E/I modules encoded within SPI-19.

Genes-Encoding T6SS Effectors					Genes-Encoding Immunity Proteins	
Gene(s)	Number of aa	Serotype-Isolate	VR	Molecular Target/Domain	Gene(s)	Transmembrane or Signal Peptide/Domain ^a^
Effectors That Target Nucleic Acids						
QS27_14110	251	*S*. Livingstone CFSAN024717	1	DNA/Tox-REase5	QS27_14115	No/Barstar
CFSAN070114_4426	1369	*S*. II 47:b:1,5 CFSAN070114	1	RNA/PAAR-RhsA-Ntox33	CFSAN070114_4425	No/DUF4279

^a^ This column indicates whether a predicted immunity protein harbors transmembrane domains (TM regions), signal peptides, or conserved protein domains.

**Table 3:** Identification of new putative T6SS E/I modules encoded within SPI-21 and a new VgrG-island.

Genes-Encoding T6SS Effectors					Genes-Encoding Immunity Proteins	
Gene(s)	Number of aa	Serotype-Isolate	VR	Molecular Target/Domain	Gene(s)	Transmembrane or Signal Peptide/Domain ^a^
**Effectors That Peptidoglycan**						
JHT71_003331 *	812	*S*. IIIb 50:r:z CLA-180-7	3	Peptidoglycan/Lysozyme-like	JHT71_003332	Signal Peptide (Sec/SPI)/No
CLA-10-1_4787	307	*S*. IIIb 61:i:z CLA-10-1	1	Peptidoglycan/Pgd-like	No	No/No
**Effectors That Target Inner Membrane**						
GI454_16755	611	*S*. IIIb 58:k:z LON-12-1	VgrG island	Phospholipids/Phospholipase D2	GI454_16750	Signal Peptide (Sec/SPII)/TPR_ DUF6396

^a^ This column indicates whether a predicted immunity protein harbors transmembrane domains (TM regions), signal peptides, or conserved protein domains. * Corresponds to truncated version of the protein.

**Table 4:** Identification of new putative anti-eukaryotic T6SS E/I modules encoded within SPI-21.

Gene(s)	Number of aa	Serotype-Isolate	Molecular Target/Domain
GI454_16220	331	*S*. IIIb 58:k:z LON-12-1	Eukaryotic V-ATPase/T6SS_Burk_ExIF
GI376_15885	985	*S*. IIIb 47:i:z FA0054	Membrane/Ttc toxin

## Data Availability

The original contributions presented in this study are included in the article/Supplementary Material. Further inquiries can be directed to the corresponding author.

## Supplementary Materials

The following supporting information can be downloaded at: https://www.mdpi.com/article/10.3390/microorganisms14061232/s1, Figure S1. Alphafold3 quality metrics of the predicted protein structures of the new candidate effectors. Table S1. *Salmonella* genomes dataset collected from Bioprojects PRJNA451499, PRJNA474529, PRJNA186035, PRJNA233520, PRJNA284726, PRJNA298359, PRJNA451499, PRJNA540977, and PRJNA560080 from the NCBI database (https://www.ncbi.nlm.nih.gov/bioproject/451499 accessed 2 May 2025, https://www.ncbi.nlm.nih.gov/bioproject/474529 accessed 2 May 2025, https://www.ncbi.nlm.nih.gov/bioproject/186035 accessed 2 May 2025, https://www.ncbi.nlm.nih.gov/bioproject/233520 accessed 2 May 2025, https://www.ncbi.nlm.nih.gov/bioproject/284726 accessed 2 May 2025, https://www.ncbi.nlm.nih.gov/bioproject/298359 accessed 2 May 2025, https://www.ncbi.nlm.nih.gov/bioproject/540977 accessed 2 May 2025, https://www.ncbi.nlm.nih.gov/bioproject/560080 accessed 2 May 2025). Table S2. Distribution of SPI-6, SPI-19, SPI-21, and VgrG island-associated T6SS effectors and candidate effectors (December 2025). Table S3. Previously identified and putative T6SS effectors encoded by *Salmonella* T6SS gene clusters.
