# Supplementary figures and images for: Type VI Secretion Systems in Salmonella Encode New Effectors with Putative Antibacterial and Anti-Eukaryotic Activities
Source: Microorganisms. 2026 May 30;14(6):1232. doi: 10.3390/microorganisms14061232 (PMC13304343; doi:10.3390/microorganisms14061232)

# QS34\_17685 - QS34\_17680

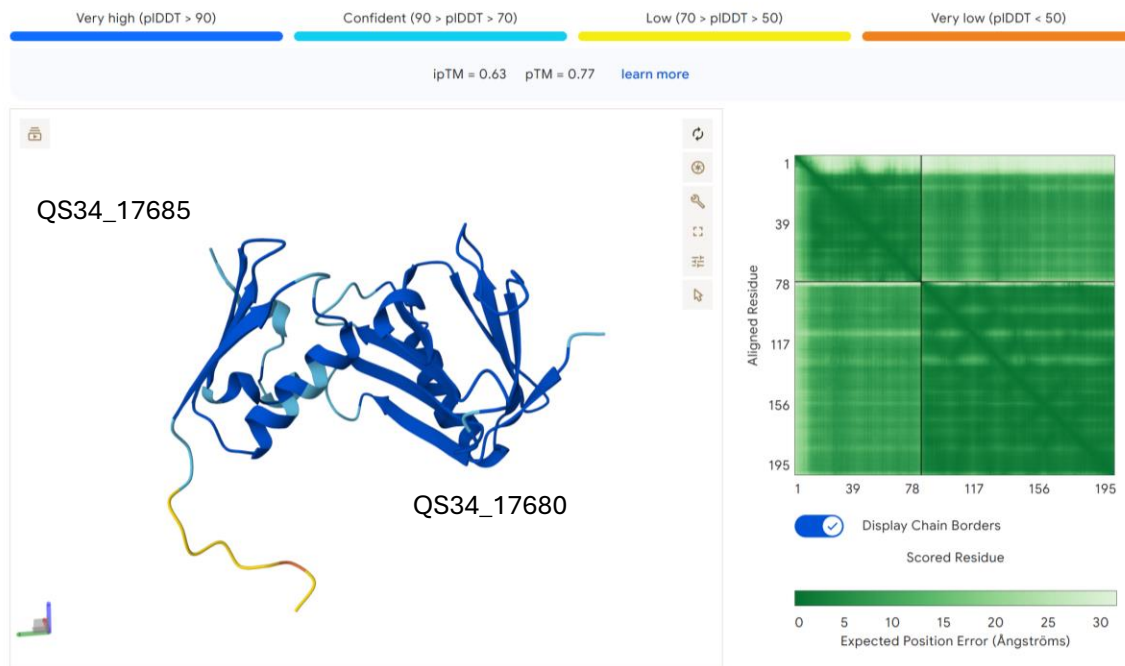

# QS27\_14110-CT - QS27\_14115

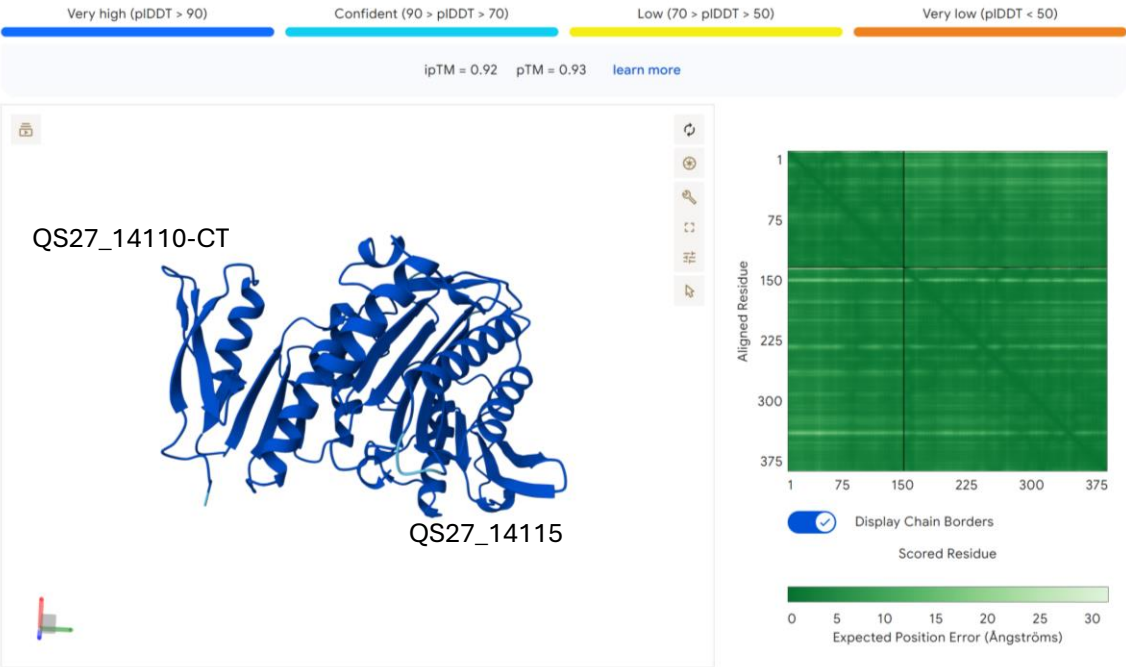

JHT71\_003331 - JHT71\_003332

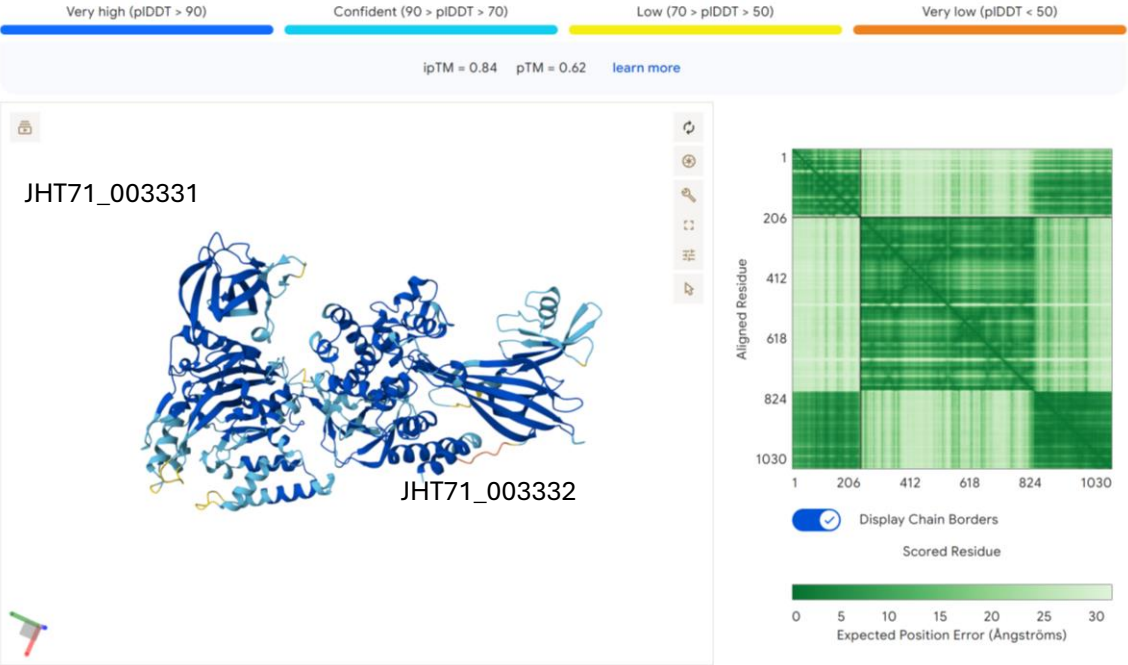

GI454\_16755 - GI454\_16750

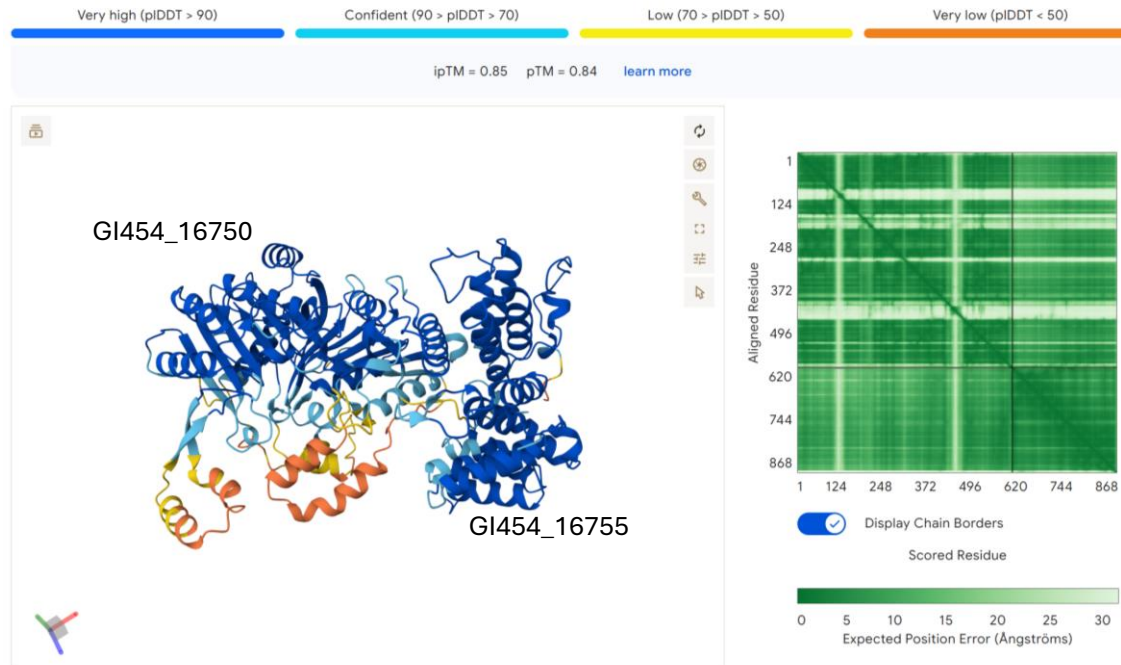

Supplement: Supplementary file 1 [file microorganisms-14-01232-s001.zip › Figure S1.pdf]
